# Annotations

**Published:** 1898-12-24

**Authors:** 


### ANNOTATIONS.

### Post-Graduate Teaching.

WE are glad to note that the new Medical Graduates, College and Polyclinic has at last so far become a reality as to have obtained suitable premises at 22 Chenies Street, W.C., and to have elected its treasurer its secretary, and its medical superintendent. Its object is "to increase the facilities offered to medical men for acquiring technical skill, and advancing their scientific and clinical knowledge," and it will seek to accomplish this object, "not only by affording instruction under its own roof, but by entering into close association with existing hospitals and medical schools." What the final outcome may be it is impossible to foresee—perhaps a very useful and important institution indeed, perhaps utter collapse. At any rate, it cannot stand still. If it succeeds it will be the germ of a very large movement, which will be vastly to the advantage of the medical profession, and will greatly improve the position of London as a centre of medical teaching, while if it fails—well, that will merely mean that the whole thing will have to be done over again, perhaps under less careful supervision, and with less complete guarantee of ethical correctness, for it is entirely unlikely that London will be allowed to remain much longer in its present anomalous position in regard to post-graduate teaching. At the meeting of the Organising Committee held last Tuesday, Mr. Jonathan Hutchinson carefully explained the scope of the proposal, and sketched out the sort of work which the institution would undertake, and the general feeling of the meeting was strongly in favour of the plans put forward. We cannot doubt that if a few good men will now and again devote an afternoon to a demonstration, and will take care to exert a controlling influence on the selection of their colleagues, such an institution will be of the utmost service to the profession.

### New Phases of Medical Journalism.

THE strict and serious business of medical journalism is threatened with innovations which tend to bring it into line with other literature of the day. Time was when men read for the sake of instruction. Possibly they do now; but they far more frequently read for amusement, and perhaps more frequently still merely to pass the time. Hence the demand for what is light, is piquant, and especially for what is short. As the result of some strange perversity, it seems to have been laid down as one of the canons of medical journalism that it must be technical, must deal with professional subjects in a professional manner, be addressed to professional readers only, and generally be so dull as not to be attractive to any except those engaged in the practice of medicine. But for the misfortune that most of the doctors have long since forgotten their Latin no doubt the purists would have had their medical journals printed in that interesting language, lest, perchance, "the laity" should read their contents. In any case it has long been held, and is still held officially, that to write on medical topics in papers which are addressed to the general public is something very bad indeed. It is, then, with some interest that we take up the prospectus of an association (limited) which is being formed for the floating of a monthly medical journal which is not only to adequately cover the field as a general medical magazine, but, "at the same time, by the publication of suitable material, serve to promote the influence of the profession among the lay community." Along with this prospectus the association forwards a list of "patrons and contributors" in which Fellows of the Royal College of Physicians stand as thick as gooseberries, even the respected president of that body being among them. What this new association means by a patron and contributor we, of course, do not know. Perhaps a contribution to its funds by the purchase of a copy may qualify a man as a "patron and contributor," and in that case we have nothing more to say. But if all the mighty men who figure in that list—men who largely rule the destinies of our profession—are really contributors in the ordinary sense of the term to a publication which, while posing as a professional organ, aims at being read by the lay community, then we think the smaller fry may fairly ask why they should be held with so tight a rein. It is not without amusement that we note another sign of that drift towards the interesting and the amusing which characterises modern medical journalism, namely, the appearance of a "short story" in the *Practitioner*. The story is a very good one, it is written by a medical man, and it has a sufficiently medical flavour about it to make it pass. Still, it is just a "short story," such as would have ornamented any ordinary magazine, and such as can be read purely as a story, without any suspicion of its clinical bearing, by any ordinary individual. The innovation is a distinctly good one, so far as that modern tendency is good which would make a journal, of whatever kind, interesting throughout—a thing of pleasure rather than a thing of toil. But it is an innovation, and one which must cause those who think a medical paper should be a sad and serious thing to shiver in their shoes. Even the *British Medical Journal*, in its programme for the coming year, proposes to deal with "archæological, historical, and literary subjects," thinking that "the addition of a little literary flavouring to the solid materials of which the *Journal* is composed may tend to make the mixture more digestible, as well as more pleasant." And who shall say that the *British Medical Journal* is not right?

### Canaries.

WE do not in this paragraph allude to the Canaries, the islands to which people fly for the cure of consumption, but to the little birds which sing songs to us in their captivity, and are, according to Dr. Tucker Wise, a cause from which consumption comes. This gentleman says that from his own observation he is of opinion that in many instances diseased cage-birds, such as canaries, communicate tuberculosis to a serious extent amongst human beings. As about 400,000 canaries are reputed to be sold every year in the United Kingdom, and as it is stated that tuberculosis is one of the most common diseases of birds, it does not seem unlikely that the canary may have a considerable influence in the distribution of tuberculous infection.

